# A zebrafish functional genomics model to investigate the role of human A20 variants in vivo

**DOI:** 10.1038/s41598-020-75917-6

**Published:** 2020-11-05

**Authors:** Daniele Cultrone, Nathan W. Zammit, Eleanor Self, Benno Postert, Jeremy Z. R. Han, Jacqueline Bailey, Joanna Warren, David R. Croucher, Kazu Kikuchi, Ozren Bogdanovic, Tatyana Chtanova, Daniel Hesselson, Shane T. Grey

**Affiliations:** 1 Immunology Division, Garvan Institute of Medical Research, 384 Victoria St, Darlinghurst, NSW 2010 Australia; 2 St Vincent’s Clinical School, The University of New South Wales Sydney, Darlinghurst, NSW 2010 Australia; 3 Diabetes Division, Garvan Institute of Medical Research, 384 Victoria St, Darlinghurst, NSW 2010 Australia; 4 The Kinghorn Cancer Centre, Garvan Institute of Medical Research, 384 Victoria St, Darlinghurst, NSW 2010 Australia; 5 Developmental and Stem Cell Biology Division, Victor Chang Cardiac Research Institute, Darlinghurst, NSW 2010 Australia; 6 Epigenetics Division, Garvan Institute of Medical Research, 384 Victoria St, Darlinghurst, NSW 2010 Australia

**Keywords:** Evolution, Genetics, Immunology, Molecular biology, Diseases, Molecular medicine

## Abstract

Germline loss-of-function variation in *TNFAIP3*, encoding A20, has been implicated in a wide variety of autoinflammatory and autoimmune conditions, with acquired somatic missense mutations linked to cancer progression. Furthermore, human sequence data reveals that the A20 locus contains ~ 400 non-synonymous coding variants, which are largely uncharacterised. The growing number of A20 coding variants with unknown function, but potential clinical impact, poses a challenge to traditional mouse-based approaches. Here we report the development of a novel functional genomics approach that utilizes a new A20-deficient zebrafish (*Danio rerio*) model to investigate the impact of *TNFAIP3* genetic variants in vivo. A20-deficient zebrafish are hyper-responsive to microbial immune activation and exhibit spontaneous early lethality. Ectopic addition of human A20 rescued A20-null zebrafish from lethality, while missense mutations at two conserved A20 residues, S381A and C243Y, reversed this protective effect. Ser381 represents a phosphorylation site important for enhancing A20 activity that is abrogated by its mutation to alanine, or by a causal C243Y mutation that triggers human autoimmune disease. These data reveal an evolutionarily conserved role for *TNFAIP3* in limiting inflammation in the vertebrate linage and show how this function is controlled by phosphorylation. They also demonstrate how a zebrafish functional genomics pipeline can be utilized to investigate the in vivo significance of medically relevant human *TNFAIP3* gene variants.

## Introduction

In mammals, the cytoplasmic ubiquitin-editing enzyme A20, encoded by the *TNFAIP3* gene, plays a key role in maintaining inflammatory homeostasis^1^ mediated by its function to inhibit NF-κB activation^2–6^. The medical importance of A20’s role in dampening NF-κB is highlighted by cases of germline A20 loss-of-function mutations in humans who present with severe autoinflammatory disease^7,8^, and by the impact of A20 deletion in mice, which results in spontaneous and widespread NF-κB activation, multi-organ inflammation and premature lethality^1,9^. The emergence of A20 mutations and coding variants as causal genetic factors driving human disease^10,11^ highlight the need to understand the functional domains of A20 that control inflammation in vivo.

A20 suppresses NF-κB signalling with inhibitory activity against key signalling molecular substrates, including TNF receptor-associated factor 6 (TRAF6)^9^, receptor interacting protein 1 (RIP1)^12,13^, and the IκB kinase complex (IKK)^14^. The A20 ovarian-tumour (OTU) domain exhibits deubiquitinating editing (DUB) activity centred on Cys103, which cleaves activating K63-linked ubiquitin chains from RIPK1, TRAF6 and NEMO to terminate NF-κB signalling^9,12,13,15^. The A20 zinc finger 4 (ZnF4) exhibits E3 ligase activity, adding K48-linked ubiquitin chains to RIPK1, triggering RIPK1 proteolysis^12,16^. In addition, the C-terminal ZnF7 domain of A20 binds linear ubiquitin to non-catalytically suppress NF-κB activity^17^. A20 is regulated at the level of gene transcription^6^, whereby NF-κB activation induces TNFAIP3 expression^18,19^ forming a negative feedback loop, and the inhibitory activities of A20 enzymatic sites are enhanced by IKKB-mediated serine phosphorylation at Ser381^20,21^.

We have previously shown that coding variants that impair A20 Ser381 phosphorylation^13^, and thereby reduce the activity of both enzymatic Cys103 and ZnF4 domains of A20^12,13,20^, exhibited a much larger in vivo effect in mice than mutations that disable the individual enzymatic sites Cys103 and ZnF4^13,21,22^. These data point towards an important role for non-catalytic Ser381 in regulating A20’s anti-inflammatory activity in vivo, however, the in vivo impact of deleting Ser381 has not been tested.

The importance of A20 in human disease, as well as the emerging functional complexities highlighted by experimental studies examining A20 mutations^13^, coupled with the discovery of new *TNFAIP3* coding variants from human genome sequencing studies (e.g. gnomAD) demonstrate the need for novel approaches to investigate A20 functional domains. We hypothesized that elucidation of A20’s conserved regions across additional species may aid the resolution of protein domains of relevance to human disease. Here we report the development of a novel functional genomics pipeline that utilizes the new A20-deficient zebrafish (*Danio rerio*) model to investigate the impact of *TNFAIP3* genetic variants in vivo. We show that A20 is essential for the maintenance of immune homeostasis and survival in zebrafish, demonstrating functionally that the emergence of A20 was essential for regulation of microbial sensing at an early junction in vertebrate evolution. Further, using this zebrafish paradigm we demonstrate how a zebrafish functional genomics approach can be utilized to investigate the in vivo significance of human *TNFAIP3* gene variants. These latter studies reveal how phosphorylation regulates A20’s evolutionary conserved anti-inflammatory activity in vivo.

## Results

### Genomic analysis of the zebrafish *tnfaip3* locus

**Figure 1:**
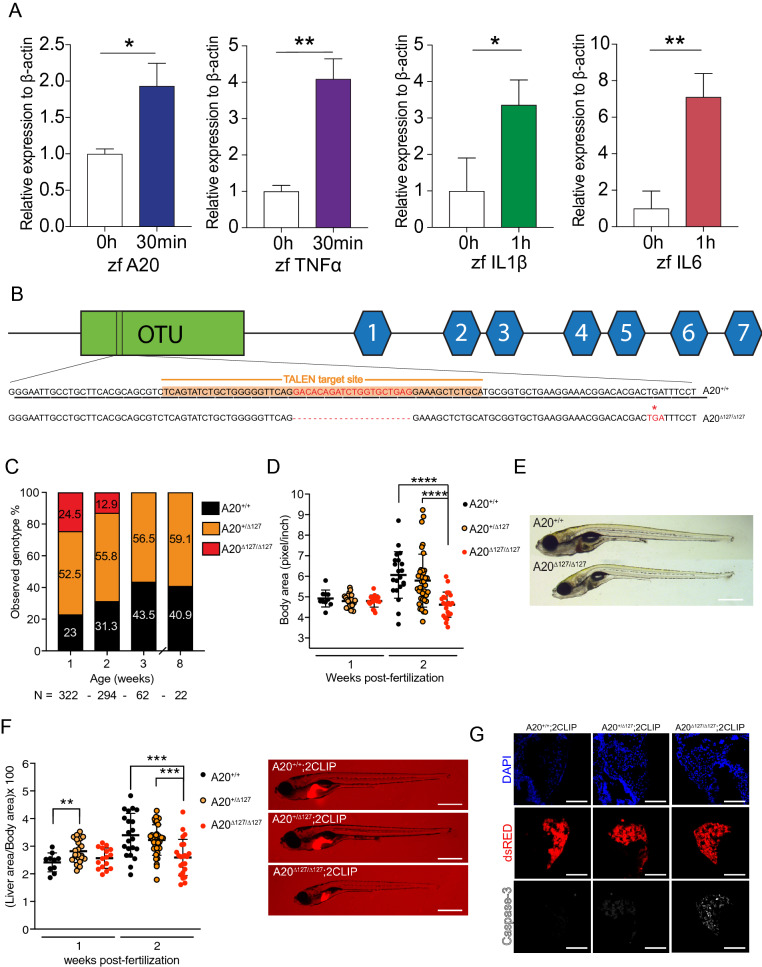
A20-deficient zebrafish fail to thrive. (**A**) Peak induction of inflammatory genes, and relative p-values. *P < 0.05, **P < 0.01. (**B**) Gene targeting of A20 highlighting the 20 bp deletion and predicted stop codon at amino acid 127, previously a Leucine. (**C**) Percentages of surviving A20^+/+^, A20^+/∆127^ and A20^∆127/∆127^ zebrafish at 1, 2, 3 and 8wpf, generated from N = 15 A20^+/∆127^ crosses. (**D**) Body area measured in (pixel/inch) per zebrafish genotype at 1 and 2 wpf. Each dot represents an individual zebrafish. Data represented as mean ± SD, P-values were determined using 1way ANOVA. ****P < 0.0001. Each group passed the normality test. (**E**) Representative brightfield image of one A20^+/+^ (top) and A20^∆127/∆127^ (bottom) zebrafish at 2 wpf. Scale bar represent 2 mm. (**F**) Liver to body ratios measured in (pixel/inch) per zebrafish genotype at 1 and 2 wpf. Each dot represents an individual zebrafish. Data represented as mean ± SD, P-values were determined using 1way ANOVA. **P < 0.01, ****P < 0.0001. Each group passed the normality test. For each genotype a representative A20^+/+^, A20^+/∆127^ and A20^∆127/∆127^ 2 wpf zebrafish is shown. The scale bars represent 2 mm. (**G**) Immuno-fluorescence sections of livers from 2CLIP reporter fish for the A20 genotypes. The scale bars represent 300 μm. Software used for this Figure: GraphPad Prism v7.0 (https://www.graphpad.com/scientific-software/prism/), Adobe Illustrator CC 2018 (https://www.adobe.com/au/products/illustrator.html), Leica LAS X (https://www.leica-microsystems.com/products/microscope-software/p/leica-application-suite/), IMARIS Image Analysis Software v8 (BITPLANE, Switzerland) (https://imaris.oxinst.com/).

Many of the mammalian components of the NF-κB signalling system^23,24^ and innate immunity^25,26^ are conserved in zebrafish making this animal model a candidate in vivo paradigm for the analysis of A20’s key functional regions. This concept was further supported by analysis of zebrafish genomic data which revealed gene order preservation of the *TNFAIP3* locus with *OLIG3* and *PERP* in zebrafish (Supplementary Fig. S1A), and a conserved and easily identifiable Topologically Associating Domain (TADs) overlapping with A20 and highly enriched in the regulatory histone mark H3K27ac (histone 3, lysine 27 acetylation) (Supplementary Fig. S1B), a hallmark of active promoters and enhancers^27,28^. Furthermore, some of these conserved H3K27c marked regions overlapped with the positions of A20 SNPs identified in human genome wide association studies (GWAS) to be linked with Crohn’s disease and Systemic lupus erythematosus (Supplementary Fig. S1A). The *tnfaip3* zebrafish orthologue^26^ encoded a predicted 762 amino acid polypeptide comprising an N-terminal ovarian tumour (OTU) domain (identity score of 94/100), including the putative catalytic cysteine at position 103 in A20’s OTU domain^29^, and seven zinc finger regions in the C-terminus (identity scores of 79/100) (Supplementary Fig. S2). The zebrafish A20 C-terminal domain also contained zinc finger 4 (ZnF4) and ZnF7 regions, which showed multiple sequence alignment scores of 91/100 and 100/100, respectively, and are thus highly homologous to functional ZnF4 and ZnF7 domains identified in mammalian studies^17,21^. In mammalian cells A20 expression is controlled at the level of transcription by the NF-κB pathway in response to microbial stimulation of TLR^19^, a pathway that represents a key component of innate immunity conserved in zebrafish^30^. Stimulation of cultured zebrafish embryos 3 days post-fertilization (dpf) with 75 µg/ml of the gram-negative bacteria product lipopolysaccharide (LPS) also resulted in the rapid induction of zebrafish (zf) zfA20 (*tnfaip3*) mRNA as well as TNF (*tnf*), zfIL-1beta (*il1b*) and zfIL-6 (*il6*) mRNA (Fig. 1A).

### Generation of a zebrafish model of A20 deficiency

Because A20 deletion in zebrafish had not been previously reported, we utilized site directed transcription activator-like effector nucleases (TALEN) to introduce a disabling mutation within exon 2 of the zebrafish A20 locus (Fig. 1B). A zebrafish line was identified that showed a 20 bp deletion in the *tnfaip3* sequence which was predicted to introduce a premature truncation at amino acid 127 (tnfaip3^gi3^, hereafter A20^∆127^). Heterozygous A20^∆127^ zebrafish were bred to homozygosity and deletion of A20 was confirmed in offspring by high-resolution melting assay (HRMA), Sanger sequencing, and analysis of PCR products that differentiate A20 wild-type alleles from A20^+/∆127^, and A20^∆127/∆127^ (Supplementary Fig. S3A–C). Heterozygous A20^∆127^ zebrafish were mated to generate A20^+/+^, A20^+/∆127^, and A20^∆127/∆127^ zebrafish.

### A20 deficiency is lethal in zebrafish

To validate the use of A20 deficient zebrafish for testing human A20 variants, we investigated the impact of zebrafish A20 deletion with respect to sentinel A20 phenotypes reported in mammals. The key mouse phenotype is premature lethality^1^ but also reported in the literature are loss of organ homeostasis, gross anatomical changes (i.e. runting), as well as hyper-responsiveness to TLR-induced NF-κB activity and heightened macrophage activity^9,31^. Accordingly, A20^+/+^, A20^+/∆127^, and A20^∆127/∆127^ zebrafish were followed through time to assess survival. A20^∆127/∆127^ zebrafish presented in the expected Mendelian ratios at 1 week post-fertilization (wpf), however, by 2wpf zebrafish homozygous for A20 deletion presented with premature lethality and no A20^∆127/∆127^ zebrafish survived to three weeks post-fertilization (Fig. 1C). Many A20^∆127/∆127^ zebrafish also exhibited gross abnormalities including reduced body size (Fig. 1D) and evident malformations of the torso (Fig. 1E). Similar to mice heterozygous for A20 deletion^1^, A20^+/∆127^ zebrafish were viable and showed no gross phenotype or lethality (Fig. 1C). Mice lacking A20 present with a rapid and early onset of systemic inflammation of the organs particularly the liver^1^. To examine the impact of A20 deletion upon organ homeostasis A20 deficient zebrafish were crossed with 2-Color Liver Insulin acinar Pancreas (2CLIP) zebrafish, which express dsRed fluorescent protein driven by the fatty acid binding protein 10 (*fabp10*) promoter in hepatocytes^32^. A20^∆127/∆127^:2CLIP zebrafish are phenotypically runted but also show a marked reduction in liver area to their body size when compared to WT zebrafish (Fig. 1F). Reduction in liver size was not due to a developmental defect as liver size was normal in 1 wpf A20^∆127/∆127^:2CLIP zebrafish. Confocal analysis of 2wpf A20^∆127/∆127^:2CLIP zebrafish livers (Fig. 1G), as well as 3-D rendering of the liver (Supplementary Videos S1 and S2), revealed a reduction in liver cellularity, and an increase in caspase-3 positive hepatocytes. We interpret these data to show that A20-deletion resulted in hepatocyte cell death and loss of organ homeostasis.

### A20 limits NF-κB activation in zebrafish

**Figure 2:**
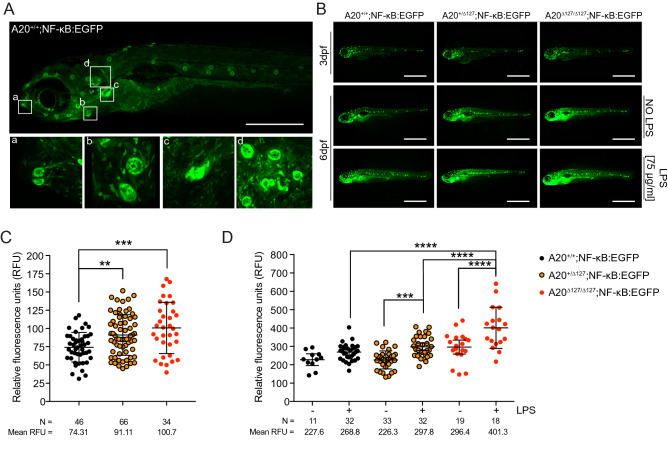
A20 is necessary to maintain inflammatory homeostasis in zebrafish. (**A**) Representative fluorescent image of a NF-κB:EGFP zebrafish at 6 dpf showing spontaneous NF-κB activity (fluorescence) in the (a) mouth, (b) lateral neuronmasts, (c) pharyngeal tooth, and (d) gills. Scale bar represents 2 mm. (**B**) Representative fluorescent images of NF-κB:EGFP zebrafish of different A20 genotypes at 3 and 6 dpf ± LPS. The scale bars represent 1 mm. (**C**, **D**) Cumulative data of relative fluorescence units of NF-κB:EGFP zebrafish by A20 genotype at; 3 dpf without LPS stimulation and 6 dpf ± LPS. Each dot represents an individual fish. Data are representative of N = 10 crosses. N per group and mean values reported below each graph. Data represented as mean ± SD, P-values were determined using 1-way ANOVA. Each group passed the normality test. **P < 0.01, ***P < 0.001, ****P < 0.0001. Software used for this Figure: GraphPad Prism v7.0 (https://www.graphpad.com/scientific-software/prism/), Adobe Illustrator CC 2018 (https://www.adobe.com/au/products/illustrator.html), Leica LAS X (https://www.leica-microsystems.com/products/microscope-software/p/leica-application-suite/).

Mice deficient for A20 are hyper-responsiveness to TLR-induced NF-κB activation^9,31^. To investigate NF-κB activation in A20 deficient zebrafish, we generated a transgenic zebrafish reporter line expressing green fluorescent protein (EGFP) under transcriptional control of a NF-κB promoter element (NF-κB*:*EGFP) and crossed these to A20^+/∆127^ zebrafish. The spontaneous responsiveness of NF-κB activation in zebrafish in vivo is a direct result of colonization by environmental microbiota^24^. As shown in Fig. 2A,B, on a wild type background the NF-κB*:EGFP* reporter demonstrates spontaneous activation. NF-κB driven fluorescence was found to be at its highest expression in those tissues that mostly interact with the external environment, namely the mouth, neuromasts, pharyngeal tooth, gills and gut (Fig. 2A). Also, NF-κB driven fluorescence increased through time (compare 3dpf A20^+/+^ zebrafish with 6dpf; Fig. 2B,C) most likely reflecting exposure to environmental microbiota^24^, and this was increased further in A20^+/∆127^ and A20^∆127/∆127^ zebrafish in a gene dose manner (Fig. 2D). Furthermore, the addition of LPS to zebrafish embryos further enhanced NF-κB activation and this was most pronounced in A20^∆127/∆127^ zebrafish (Fig. 2D).

### Zebrafish A20-deficient macrophages are hyper-responsive to inflammatory triggers

**Figure 3:**
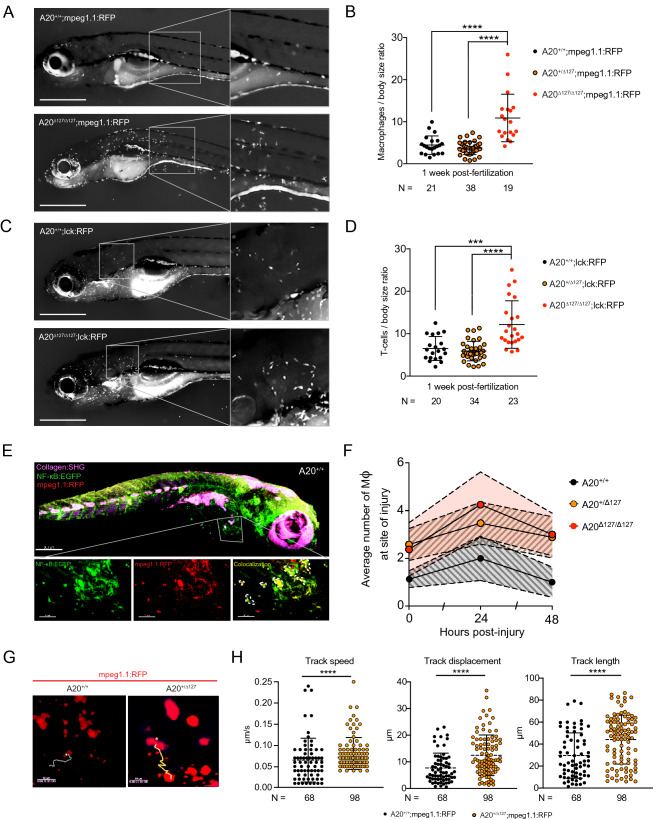
Impact of A20 deletion on macrophage activity in zebrafish. (**A**) Representative fluorescent image of A20^+/+^;mpeg1.1:RFP (top) and A20^∆127/∆127^;mpeg1.1:RFP (bottom) zebrafish at 1 wpf. (**B**) Number of macrophages counted from images of zebrafish by A20 genotype as in (**A**). Data represented as mean ± SD, P-values were determined using Whelch’s t-test. ****P < 0.0001. (**C**) Representative fluorescent image of A20^+/+^;lck:RFP (top) and A20^∆127/∆127^;lck:RFP (bottom) zebrafish at 1 wpf. (**D**) Number of dermal T lymphocytes counted from images of zebrafish by A20 genotype as in (**C**). Data represented as mean ± SD, P-values were determined using Whelch’s t-test. ***P < 0.001, ****P < 0.0001. (**E**) 3-D rendering of a Two-Photon microscope tilemap of a double-reporter (NF-κB:EGFP;mpeg1.1:RFP) A20^+/+^ zebrafish. The image shows three channels: Collagen (in magenta) generated through Second Harmonic, NF-κB (in green) generated though EGFP fluorescence and mpeg1.1 (in red) generated through RFP fluorescence. The accompanying video (Supplementary Video S3) navigates through the 3D rendering. IMARIS Image Analysis Software v8 (BITPLANE, Switzerland) (https://imaris.oxinst.com/). (**F**) Average number of macrophages at the site of injury measured at 24 and 48 h post-injury. Error bands are SEM. Area under the curve at 24 hpi was significant for A20^+/+^ x A20^+/∆127^ (*p < 0.05) and A20^+/+^ x A20^∆127/∆127^ (*p < 0.05). (**G**) Screenshots of a Two-Photon z-stack 15 min time-lapse track path of an A20^+/+^;mpeg1.1:RFP and A20^∆127/∆127^;mpeg1.1:RFP zebrafish in the resting state. The tracks show the path taken by a representative macrophage expressing RFP. The chosen macrophages are the closest to the average tracking data. (**H**) Macrophages from mpeg1.1:RFP positive zebrafish were imaged using in vivo two-photon microscopy and their track speed, track displacement and track length were quantified and used to determine a ‘meandering index’. Data collected from 4 A20^+/+^ and 3 A20^+/∆127^ comprising of 68 and 98 macrophages tracks respectively. Data represented as mean ± SD, P-values were determined using Mann–Whitney t-test. ****P < 0.0001. Software used for this Figure: GraphPad Prism v7.0 (https://www.graphpad.com/scientific-software/prism/), Adobe Illustrator CC 2018 (https://www.adobe.com/au/products/illustrator.html), Leica LAS X (https://www.leica-microsystems.com/products/microscope-software/p/leica-application-suite/), IMARIS Image Analysis Software v8 (BITPLANE, Switzerland) (https://imaris.oxinst.com/).

A20 loss of function mutations result in macrophage hyper responsiveness to microbial stimulation and spontaneous inflammasome activation^9,13,17^. Tissue macrophages play a key role in sensing pathogenic microbiota in zebrafish. To investigate the impact of A20 deletion on macrophage homeostasis, heterozygous A20^+/∆127^ zebrafish were crossed to a macrophage reporter line whereby RFP is expressed under the control of the promotor of macrophage-expressed gene 1.1 (*mpeg1.1*). The number of macrophages detected in the skin was ~ 2- to 4-fold higher in A20^∆127/∆127^ zebrafish when compared to their wild type counterparts (Fig. 3A,B). We also created a A20^∆127/∆127^ T cell reporter line whereby RFP is expressed under the control of the lymphocyte-specific protein tyrosine kinase promotor lck, this reporter line showed that A20 deletion also led to increased numbers of dermal T cells (Fig. 3C,D). In addition to increased numbers, the majority of dermal macrophages in A20^∆127/∆127^ zebrafish exhibited dendritic extensions (Fig. 3A magnification) indicative of an activated state^33,34^. Imaging of double reporter (NF-κB*:*EGFP x mpeg1.1:RFP) WT zebrafish by two-photon microscopy (Fig. 3E, Supplementary Video S3) showed co-localisation of NF-κB activation with MPEG1 reporter expression further highlighting activation of dermal macrophages in A20^∆127/∆127^ zebrafish. This was also made further apparent by histological analysis through transverse sections of the cranium, which revealed dense infiltration of mpeg1.1 positive cells close to the dermal surface (Supplementary Fig. S4). From these cranial sections it could be seen that the position of the mpeg1.1 positive cells corresponded with cells at the dermal surface exhibiting bright NF-κB*:*EGFP reporter activity.

Recruitment to sites of inflammation constitute important physiological roles for the macrophage immune compartment. Using a tail clip wound model^35^ we found that both A20^+/∆127^ and A20^∆127/∆127^ zebrafish showed an increased recruitment of macrophages at the wound interface through time (Fig. 3F). Interestingly, this analysis revealed a macrophage phenotype for heterozygous A20^+/∆127^ zebrafish post challenge whereas we did not observe a change in A20^+/∆127^ macrophage numbers in the steady state (Fig. 3B). To investigate this further, we used two-photon imaging of single mpeg1.1:RFP reporter A20^+/∆127^ zebrafish to track individual macrophage cell movement in vivo in real time (Fig. 3G,H, supplemented by Supplementary Video S4). This analysis revealed that A20^+/∆127^ macrophages exhibited increased cell movement in the steady state (track length:displacement ratio = 3.95 for WT macrophages versus 4.55 for A20^+/∆127^ macrophages), and was attributed to increases in track speed, track displacement and track length (Fig. 3H).

### Human A20 rescues A20^∆127/∆127^ zebrafish from lethality

**Figure 4:**
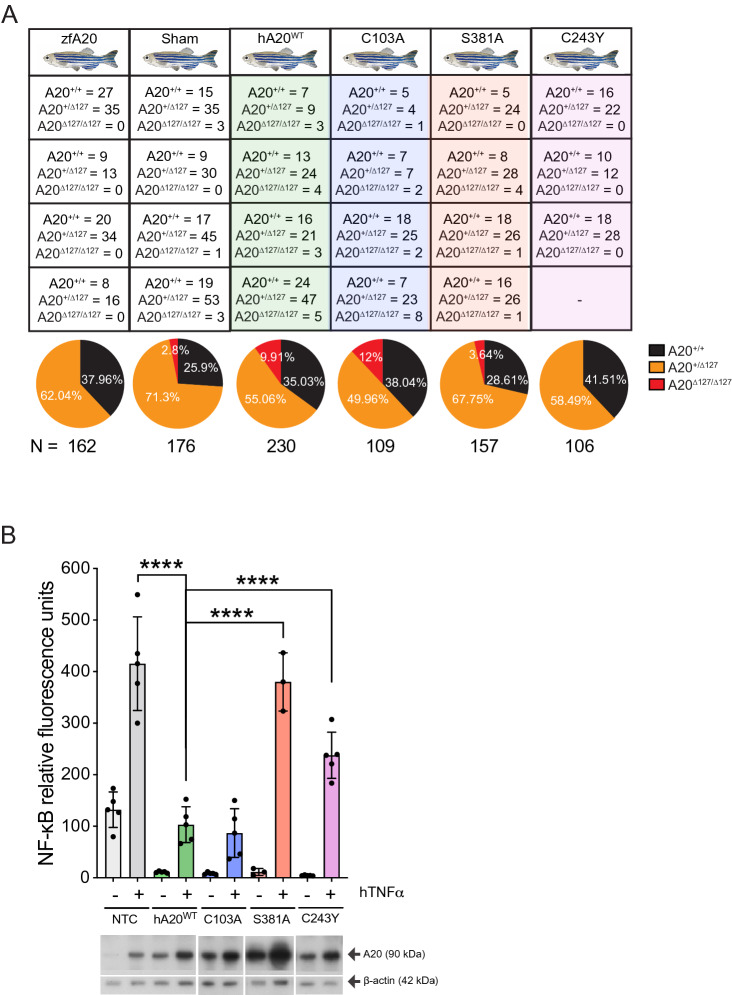
The impact of human A20 mutants on A20^∆127/∆127^ zebrafish survival. (**A**) Survival data for A20^+/+^, A20^+/∆127^ and A20^∆127/∆127^ zebrafish at 3 wpf (zfA20), mock injected (Sham) or with ectopic administration of wild-type human A20 (hA20^WT^), C103A, S381A or C243Y mutants. Numbers indicate number of genotyped survivors expressing the respective human gene variant. Each box indicates the survivors from an independent experiment. Survival frequency data is represented in the pie chart. N indicates the total number of zebrafish embryos injected for each A20 mutant. Statistical difference was observed between zfA20 and C103A injected/rescued fish (*p < 0.05) and between C103A and C243Y injected fish (*p < 0.05). (**B**) NF-κB luciferase assay for HEK293 cells transiently non-transfected (NTC), or co-transfected with wild-type human A20 (hA20^WT^) or the C103A, S381A or C243Y mutant and treated with or without hTNFα. Data represented as mean ± SD. P-values were determined using ANOVA. Each dot represents an individual experiment and are representative of N = 3–5 experiments per A20 mutant. Below is shown a representative Western blot for each A20 mutant. The displayed gel has been cropped to improve clarity and conciseness of the figure. Software used for this Figure: GraphPad Prism v7.0 (https://www.graphpad.com/scientific-software/prism/), Adobe Illustrator CC 2018 (https://www.adobe.com/au/products/illustrator.html), Bio-rad ChemiDoc v6 (https://www.bio-rad.com/en-au/product/chemidoc-imaging-system?ID=OI91XQ15).

The phenotypic similarities between A20-deficient zebrafish and those reported for A20-deficient mice^1,9,17^, as well as the profound inflammatory syndromes reported for human subjects with heterozygous A20 deficiency^8^, supported utilizing zebrafish as a model organism to investigate the role of A20 protein domains in regulating its function in vivo. For our functional analysis pipeline, we determined that rescue of A20^∆127/∆127^ zebrafish from lethality would provide the most robust readout for loss or gain of function phenotypes in various A20 genetic variants. For the functional analysis, zebrafish eggs derived from A20^+/∆127^ × A20^+/∆127^ crosses were ectopically injected with wild-type human (h)A20 expressed in the construct Ubb-hA20-P2A-EGFP that also expresses GFP. The low integration Ubb-hA20-P2A-EGFP construct was chosen to limit potential off-target effects as well as false positives^36–38^. The zebrafish eggs were injected in a blinded fashion with the Ubb-hA20-P2A-EGFP construct and were assessed for the presence of GFP fluorescence (Supplementary Fig. S5A). Human A20 expression in injected embryos was confirmed by mRNA analysis (Supplementary Fig. S5B). At 1 wpf A20^∆127/∆127^ zebrafish presented in the expected mendelian ratio of ~ 25% of genotypes (Fig. 1C) but later exhibited a highly penetrant lethal phenotype with no survivors at 3wpf (Fig. 4A). In contrast, with ectopic expression of hA20, A20^∆127/∆127^ zebrafish now comprised 9.91% of the total of all genotypes at 3wpf (Fig. 4A). Further, all surviving A20^∆127/∆127^ zebrafish were positive for EGFP expression derived from the Ubb-hA20-P2A-EGFP construct (Supplementary Fig. S5C). Given an expected Mendelian ratio of 25%, ectopic expression of hA20 rescued ~ 40% of A20^∆127/∆127^ zebrafish from lethality highlighting the strong evolutionary conservation for A20’s function across the veterbrate lineage.

### Alanine substitution of Ser381 and the human mutation C243Y impairs A20’s in vivo function

We previously demonstrated that a series of A20 coding variants, all within A20’s OTU domain, showed a strong correlation between a graded loss of A20’s NF-κB inhibitory function and an increasing hyper-inflammatory in vivo phenotype with reduced phosphorylation at Ser381^13^. The coding variant C243Y which showed the strongest in vivo phenotype and caused the greatest reduction in Ser381 phosphorylation was originally identified in a four generation Japanese family with hereditary autoinflammatory disease^7^. In vitro biochemical studies show that IκB kinase beta (IKKβ)-dependent phosphorylation at Ser381 is required for A20’s optimal NF-κB and JNK suppressive function^13,20,39^ (Fig. 4B; Supplementary Fig. S5D,E). Both Ser381 and Cys243 are conserved in zebrafish (Supplementary Fig. S2) allowing us to test the impact of alanine substitution at Ser381, and the impact of the disease causing variant C243Y on the ability of human A20 to rescue A20^∆127/∆127^ zebrafish from lethality.

As shown in Fig. 4A, ectopic injection of either S381A or C243Y expressing constructs into A20^∆127/∆127^ embryos rescued less fish from death compared to A20^∆127/∆127^ embryos that received wild-type hA20-expressing constructs. Specificity for the impact of S381A and C243Y on A20’s in vivo function was shown by ectopic injection of another OTU domain mutant, namely the C103A DUB mutant^13,21,22^, which niether impaired A20’s ability to rescue A20^∆127/∆127^ embryos from lethality, nor A20’s ability to block NF-κB or JNK signalling in vitro (Fig. 4A,B; Supplementary Fig. S5D,E). The in vivo rescue properties of individual A20 mutants did not relate to differences in protein stability, as shown by Western blot analysis of A20 protein levels when expressed in cell lines (Fig. 4B)^13^, nor differences in the levels of transgene expression in vivo as measured by the levels of GFP fluorescence which is co-expressed with A20 in the Ubb-hA20-P2A-EGFP construct (Supplementary Fig. S5A).

## Discussion

A20 inhibits NF-κB activation in mammals in response to TLR signals^1,9^, and uncontrolled NF-κB activation results in excessive inflammation and lethality^40^, yet there is little information regarding the in vivo functional role of A20 beyond mammalian models. An A20-like protein sequence has been identified in the purple sea urchin and amphioxus^41^ and in the lamprey genomes^42^ and here we identify a functional A20 homologue in zebrafish. In contrast, we did not find a homologous A20 sequence in the genomes of lower invertebrates including *Drosophila*. Similar to mammalian cells, and as shown here for zebrafish, A20 expression in amphioxus is also regulated by LPS^19,41^ and in both amphioxus and mammalian cells A20 modulates NF-κB signalling via a ubiquitin editing function^13,21,41^. However, the in vivo functional significance of A20 in amphioxus has not been tested^41^. Reminiscent of mouse data^1,9^, we found that A20 deficiency in zebrafish resulted in spontaneous hyper-responsiveness to environmental microbiota with early lethality. Remarkably re-introduction of human A20 could rescue a proportion of A20-deficient zebrafish from lethality. These data highlight the strong functional evolutionary conservation for A20 in vertebrates, suggesting that A20 arose early during chordate evolution to regulate inflammatory homeostasis. As the major components of NF-κB signalling in mammals are conserved to *Drosophila*^13,43^ it is unclear as to why A20-dependent control of NF-κB signalling was required for chordate evolution, but further studies to investigate A20 function in the purple sea urchin would be informative*.* In mammalian cells A20 has been shown to regulate diverse signalling cascades that control inflammation and cell death in both hematopoietic and non-hematopoietic cell lineages^10,44^. Further investigation of A20’s function in non-mammalian systems like zebrafish will reveal to what extent these critical mammalian functions extend to other vertebrates and aid our understanding of the evolutionary significance of A20.

Gene targeting in mice to either introduce an alanine at Cys103 (C103A) that eliminates biochemical OTU DUB activity^21^, or a mutation that destroys the ZnF4 E3 ligase activity of A20^22^, resulted in surprisingly little or no impact on lipopolysaccharide (LPS) responses and inflammatory homeostasis in vivo^22,45^, contrasting the lethal hyper-inflammatory phenotype of A20-deficient mice^1,9^. These findings indicate compensatory activity of the Cys103 and ZnF4 catalytic domains towards each other, and redundancy in the mechanism by which the OTU domain regulates NF-κB activation, which may include both catalytic and non-catalytic roles^12,14^. Previous biochemical studies have shown that A20’s NF-κB inhibitory activity requires phosphorylation at Ser381 by IκB-kinase^20,21^. We previously provided evidence for the importance of Ser381 in vivo^13^. We demonstrated how a series of A20 coding variants that cause a decrease in phosphorylation at Ser381 also decrease A20’s NF-κB inhibitory activity in a graded manner, with a corresponding reduction in the ability of A20 to control inflammatory homeostasis in vivo. Such coding variants can have medical relevance, they may be beneficial by increasing protective immunity to pathogens^13^, however increased tissue inflammation may also be detrimental in situations including autoimmune disease^7^ and organ transplantation^46^.

Here, using a new zebrafish functional genomics approach, we show evidence that Ser381 phosphorylation is needed for A20’s optimal anti-inflammatory function in vivo by directly targeting Ser381. Targeted mutation of Ser381 impaired the ability of A20 to rescue A20^∆127/∆127^ zebrafish from lethality. Reduced Ser381 phosphorylation disables both the OTU DUB and ZnF4 ubiquitin editing functions of A20^13^. This could explain the highly penetrant phenotype seen for both zebrafish and mice^13^ carrying variants that impact Ser381 when compared to the relatively mild phenotypes observed in mice carrying mutations that disable the individual catalytic domains^22,45^. Further studies are required to understand how phosphorylation at Ser381 impacts A20’s function but one possibility is that phosphorylation causes changes to A20 structure facilitating interactions between A20’s distinct ubiquitin binding and ubiquitin editing domains with ubiquitin. Indeed, two studies highlight the importance of ubiquitin binding domains (i.e. ZF4 and ZF7) in preventing inflammatory disease^47,48^. We predict that access to ubiquitin by these domains is regulated by A20 phosphorylation^13^. Understanding how specific molecular changes in A20 effect function and cellular phenotypes may provide a pathway to understand the seemingly broad association of A20 to many different tissue specific autoimmune diseases identified through GWAS^10^ and the broad clinical phenotypes emerging in patients identified with A20 haploinsufficiency^41,49,50^.

A20 deletion results in severe autoinflammatory disease in humans^31,49–52^. The severe impact of mutating Ser381 on A20 function shown here, and the discovery of an ever-increasing number of new A20 coding variants through human sequencing studies^13^, raise the question as to the existence of further human coding variants, which may present with highly penetrant phenotypes and contribute to disease. The conservation of A20 in zebrafish allows us to construct a functional genomics pipeline to investigate the impact of medically and scientifically important human A20 coding variants in vivo. The discovery of the cysteine to tyrosine substitution at position 243 (C243Y) in a four generation Japanese family with a Behcet’s like disease^7^, together with the high level of sequence homology across the mammalian and zebrafish OTU domains which included this residue, provided an opportunity to test the sensitivity of the zebrafish pipeline. In this zebrafish model the C243Y mutation caused a reduction in A20’s ability to rescue homozygous A20-null zebrafish from lethality. This in vivo proof of concept data in the zebrafish model showing that C243Y is a reduction of function variant is supported by the human genetic data^7^, as well as biochemical and mouse data^13^. A20 variants like C243Y that modify phosphorylation at Ser381 therefore represent a novel class of variants with medically relevant disease-causing potential.

We highlight the potential of the zebrafish system to advance our knowledge of medically significant A20 gene variants by taking advantage of regions of high homology. In addition to shedding light on the functional significance of A20 coding variants, the conservation of the zebrafish A20 genomic locus may also aid the elucidation of non-coding variants. GWAS studies associate A20 with multiple autoimmune conditions and many identified SNPs are non-coding^10^. Analysis of conserved trans regulatory regions in the A20 locus marked by active methylation marks revealed an overlap with two SNPs associated with Crohn’s disease (rs7753394; rs7773904) (GRCh38) and one associated with Systemic lupus erythematosus (rs10499197)^53–55^. As these SNP’s lay in putative enhancer regions, they may alter A20 expression levels thereby contributing to disease. The development of a novel functional genomics pipeline that utilizes A20-deficient zebrafish model provides a new tool to investigate the impact of *TNFAIP3* genetic variants in vivo. Understanding the underlying genetic code of A20 will have relevance for understanding human disease mechanisms and the development of targeted drug therapies. This same approach could be utilized to increase understanding of the impact of human genetic variation for other highly conserved genes.

## Materials and methods

### Zebrafish

Fertilized eggs were collected from zebrafish (*Danio rerio*) group matings and incubated at 28.5 °C at a density < 60 embryos in ~ 25 mL E3 egg water. Adult zebrafish were maintained under standard conditions at 28.5 °C and pH 7–8 at a density of 5–10 fish L^−1^ under a photoperiodic cycle of 14/10 light/dark hours. Genotyping was performed by fin clipping and HotSHOT DNA extraction^56,57^ followed by High Resolution Melting assay. Animal studies were approved by the Garvan/St Vincent's Animal Ethics Committee. All procedures performed complied with the Australian Code of Practice for Care and Use of Animals for Scientific Purposes.

### Genomic structure of A20

HiC data (40 Kb resolution) corresponding to H1 cells were visualized in the 3D genome browser^58^ (hg19 genome assembly). Layered H3K27ac ChIP-seq data from seven cell lines (GM12878, H1-hESC, HSMM, HUVEC, K562, NHEK, NHLF), available through the UCSC genome browser^59^ were generated by the ENCODE consortium (2012). Zebrafish 24hpf embryo H3K27ac ChIP-seq reads were mapped by Bowtie^60^ to unique genomic positions, followed by duplicate removal. Mapped ChIP-seq reads were visualized in WIG format as previously described^61^ utilizing the UCSC genome browser.

### *Tnfaip3* knockout line

A pair of TALENs targeting zebrafish *tnfaip3* (A20) exon 2 were generated using “PLATINUM Gate TALEN Kit” (Addgene, #1000000043). 0.2 ng of mRNA encoding the TALEN pair was delivered into the cytoplasm of wild-type zebrafish embryos at the one-cell stage. Founders were genotyped using the following primers: zfA20_Fw: AGTATCTGCTGGGGGTTCAGGA and zfA20_Rev: CACCGCATGCAGAGCTT. Subsequently a founder line was identified harbouring a 20 bp deletion in exon 2 with a predicted stop codon at amino acid 127. The *tnfaip3*^dGI4^ A20-deficient line was maintained in the heterozygous state (tnfaip3^∆127^/tnfaip3^+^) and crossed to the zebrafish reporter lines described below.

### Zebrafish reporter lines

The lck:RFP (TgBAC(*lck*:RFP)^vcc4^) and mpeg1.1:RFP (TgBAC(*mpeg1.1*:RFP)^vcc7^) reporter lines have been described previously. The lymphocyte protein tyrosine kinase (*lck*) promoter targets gene expression to T cells whereas macrophage expressed gene 1.1 (*mpeg1.1*) promoter targets gene expression to macrophages. The Tg(ins:dsRed)^m1081^;Tg(fabp10:dsRed;ela3l:GFP)^gz12^ line, known as 2-Color Liver Insulin acinar Pancreas (2CLIP) fish line, has been described previously^62^. This fish express dsRed fluorescent protein both in the hepatocytes, driven by the fatty acid binding protein 10 (*fabp10* gene), and in the islets of the endocrine pancreas, driven by the insulin promoter (*insulin* gene). The exocrine pancreas instead expresses GFP driven by the pancreas specific transcriptor factor 1a (*ptf1a* gene). We generated the NF-κB reporter line Tg(*NF-κB, c-FOS*:EGFP)^GI5^ zebrafish line was generated using the Tol2 system to insert a DNA fragment containing six repeats of an NF-κB binding site in tandem with a minimal c-Fos promoter that drive the expression of EGFP, followed by an SV40 polyadenylation signal sequence (SV40pA), this construct has been previously reported^24^.

### Fluorescence quantification through ImageJ

Live zebrafish were placed in a gel solution (1 × E3 solution, 3% methyl cellulose, 5% Tricaine), in a 150 mm petri dish lid. Fish were placed on the left side up. Images were processed using ImageJ (v.1.50b). In the case of the NF-κB:EGFP line fluorescence measurements, an outline of the fish was drawn by hand, excluding the yolk sack due to its auto fluorescence. Measurement values of area (pixel/inch) and integrated density (area × mean grey value) were recorded for each of the measured fish. Background value was subtracted to the fish intensity value.

### NF-κB and JNK reporter assays

The NF-κB reporter assay was conducted exactly as described previously^19^. In brief, human Embryonic Kidney 293T (HEK293T) cells^63^ were cultured in Dulbecco's Modified Eagle's medium (DMEM) and incubated at 37 °C in 5% CO_2_ and kept at a passage number between 21–40 for experiments. Cells were transfected using Lipofectamine 2000 (ThermoScientific #11668019) with β-galactosidase (β-gal) and NF-κB.Luc with either ‘empty’ pcDNA3.1 vector (NTC; non template control) or pcDNA3.1 encoding wild-type human-A20 or either the A20 mutant Cys103Ala or Ser38Ala. HEK293T cells were stimulated with 300 U/ml of recombinant human TNFα (RandD Systems, Minneapolis, MN; #210-TA) for 8 h. To assess the effect of A20 mutants on JNK signalling, HEK293T cells which constitutively expresses mCherry Kinase Translocation Reporter (mCherryKTR) which translocates from the nucleus to the cytoplasm when phosphorylated by JNK were used (de la Cova, Townley et al. 2017). JNK activity was determined by measuring the ratio between nucleus and cytoplasmic mCherry in cells transiently transfected with human-A20 mutants (using JetPRIME reagent) following human TNFα stimulation. Each experiment included automated cell imaging (Thermo Scientific Cellomics Arrayscan machine) of ~ 150,000 to ~ 350,000 individual cells per well in duplicate wells over a period of 60 min (5 min intervals).

### Generation of A20 mutants for ectopic expression in zebrafish

Three A20 mutants were generated for this study. Mutations were introduced into cDNA encoding full length human A20 by site directed mutagenesis to cause an alanine substitution at position Cys103 (C103A), a second clone at Ser381 (S381A) and a third clone a Tyrosine at position Cys243 (C243Y). Wild type A20 or mutant C103A, S381A or C243Y A20-cDNA were cloned into the expression cassette Ubb-[A20 insert]-P2A-EGFP and ~ 100 ng/μl of DNA was co-injected with I-SceI meganuclease to promote random integration in the zebrafish genome. Glass needles for zebrafish eggs injections were prepared from glass capillary tubes with filament (ø1 mm × 90 mm) (WPI #1B100F-3) using a NARISHIGE dual-stage micropipette puller PC-10 machine. The injections were all performed between the one and two-cell stage of development. The pUbb-hA20-P2A-EGFP was generated by modifying the published plasmid pUbb-iCre-EGFP^64^ in which iCre is fused to EGFP via a P2A sequence which sits downstream of the promoter of the zebrafish *ubb* gene. The entire cassette is flanked with with I-SceI (NEB, Ipswich, MA) sites. To generate pUbb-hA20-P2A-EGFP, iCre cDNA of pUbb-iCre-EGFP was replaced with hA20 cDNA using restriction enzyme digest cloning, and the resultant plasmid was co-injected with I-SceI into one-cell-stage embryos.

### Two-photon intravital microscopy

Two-photon imaging was performed using an upright Zeiss 7MP two-photon microscope (Carl Zeiss) with a W Plan-Apochromat 20 × /1.0 DIC (UV) Vis-IR water immersion objective. Four external NDDs were used to detect blue (SP 485), green (BP 500–550), red (BP 565–610) and far red (BP 640–710). High repetition rate femtosecond pulsed excitation was provided by a Chameleon Vision II Ti:Sa laser (Coherent Scientific) with 690–1064 nm tuning range. We acquired 3 μm z-steps at 512 × 512 pixels and resolution 0.83 μm/pixel at a frame rate of 10 fps and dwell time of 1.27 μs/pixel using bidirectional scanning. Intravital two-photon microscopy was performed by embedding 1 wpf zebrafish in 1% low melting point agarose in E3 water and 0.4% Tricaine to limit the overall tissue drifting and motility of the fish.

### Image processing and data analysis

Raw image files were processed using Imaris (Bitplane) software. A Gaussian filter was applied to reduce background noise. Tracking was performed using Imaris spot detection function to locate the centroid of cells. Motility parameters such as cell displacement (or track length calculated as the total length of displacements within the track) were obtained using Imaris Statistics function.

### Statistical analysis

Data were analysed as appropriate using a nonparametric Mann–Whitney test, a Whelch t-test, Area under the curve (AUC) or a one-way ANOVA test. Statistical analyses were carried out using GraphPad Prism v7.0. In all cases, the significance threshold was set at *p* ≤ 0.05. For clarity, only significant comparisons were reported on each graph.

## Supplementary information

Supplementary Figures.

Supplementary Video 1.

Supplementary Video 2.

Supplementary Video 3.

Supplementary Video 4.

Supplementary Legends.
