# Supplementary material for: A zebrafish functional genomics model to investigate the role of human A20 variants in vivo
Source: Sci Rep. 2020 Nov 5;10:19085. doi: 10.1038/s41598-020-75917-6 (PMC7644770; doi:10.1038/s41598-020-75917-6)
Supplement: Supplementary file 1 — Supplementary Figures. [file 41598_2020_75917_MOESM1_ESM.pdf]

A zebrafish functional genomics model to investigate the role of human A20 variants in vivo

Authors:
Cultrone, Daniele[1,2]; Zammit, W. Nathan[1,2]; Self, Eleanor[1,2]; Postert, Benno[2,3]; Han, Jeremy ZR[2,4]; Bailey, Jacqueline[1,2]; Warren, Joanna[1,2]; Croucher, David R[2,4]; Kikuchi, Kazu[2,5]; Bogdanovic, Ozren[2,6]; Chtanova, Tatyana[1,2]; Hesselson, Daniel[2,3]; Grey, Shane T. [1,2]†

Affiliations:
[1]Immunology Division; [3]Diabetes Division; [4]The Kinghorn Cancer Centre; [6]Epigenetics Division; Garvan Institute of Medical Research, 384 Victoria St, Darlinghurst, New South Wales, 2010 Australia
[2]St Vincent’s Clinical School, The University of New South Wales Sydney, New South Wales, 2010 Australia.
[5]Developmental and Stem Cell Biology Division, Victor Chang Cardiac Research Institute, Darlinghurst, NSW 2010, Australia

†Corresponding Author and Lead Contact: Email s.grey@garvan.org.au (S.T.G.)

Running title: An evolutionary conserved role for TNFAIP3 to limit inflammation in vertebrates

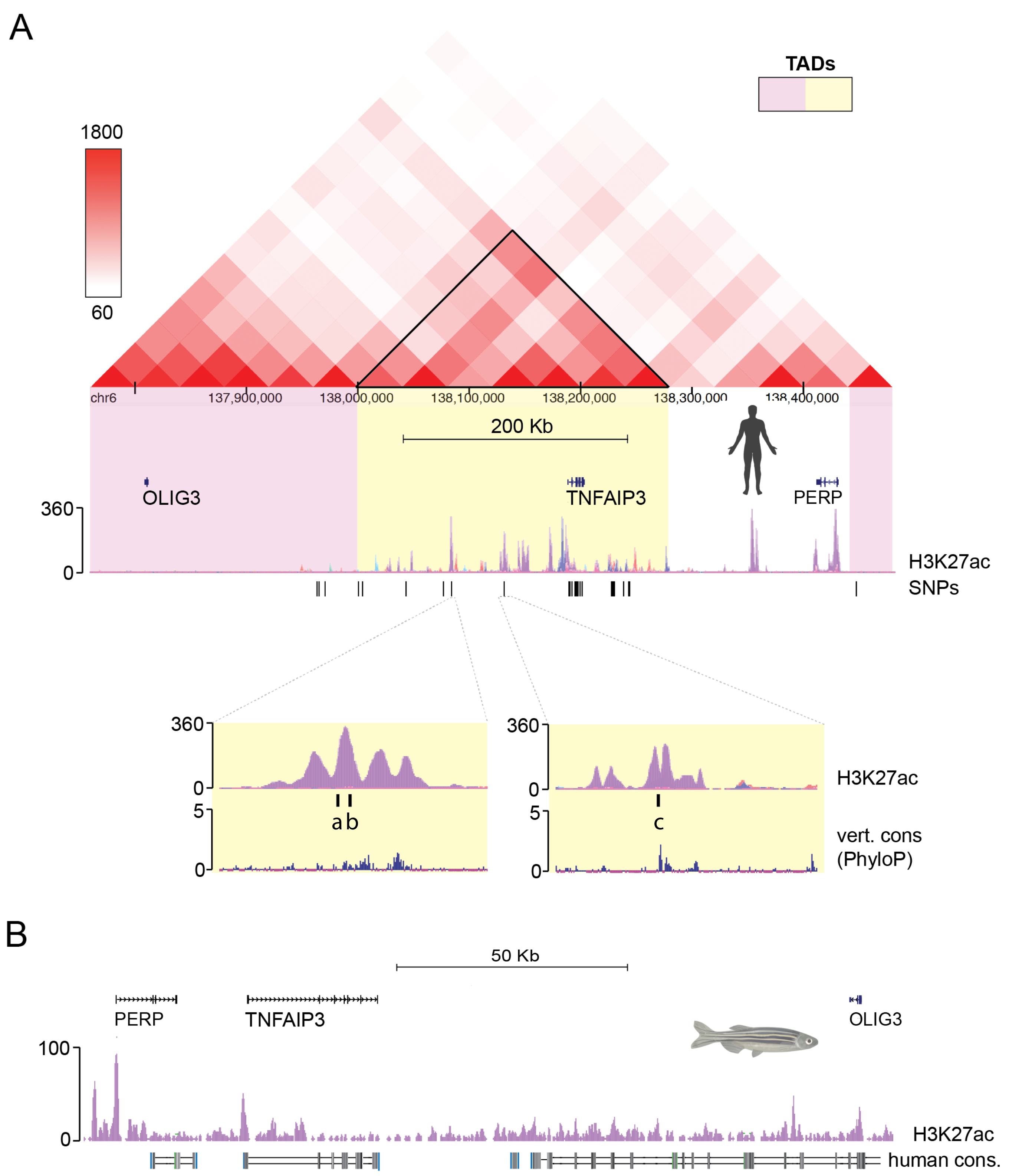

A
TADs
1800
60
chr6
137,900,000
138,000,000
138,100,000
138,200,000
138,300,000
138,400,000
200 Kb
OLIG3
TNFAIP3
PERP
360
0
H3K27ac
SNPs
360
0
5
0
a b
360
0
5
0
c
H3K27ac
vert. cons
(PhyloP)
B
50 Kb
PERP
TNFAIP3
OLIG3
100
0
H3K27ac
human cons.

Figure S1

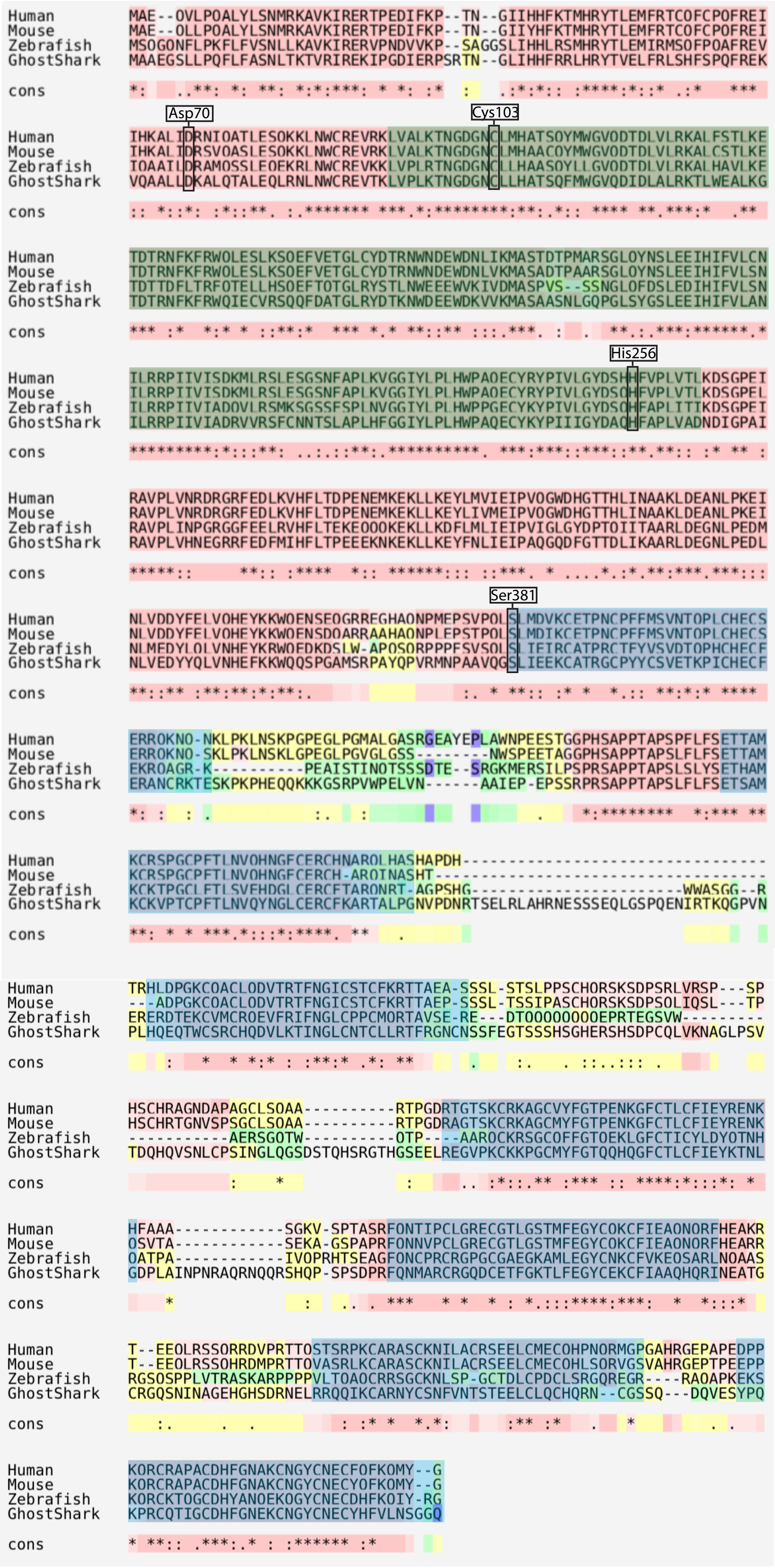
Asp70
Cys103
His256
Ser381
Human
Mouse
Zebrafish
GhostShark
cons

Figure S2

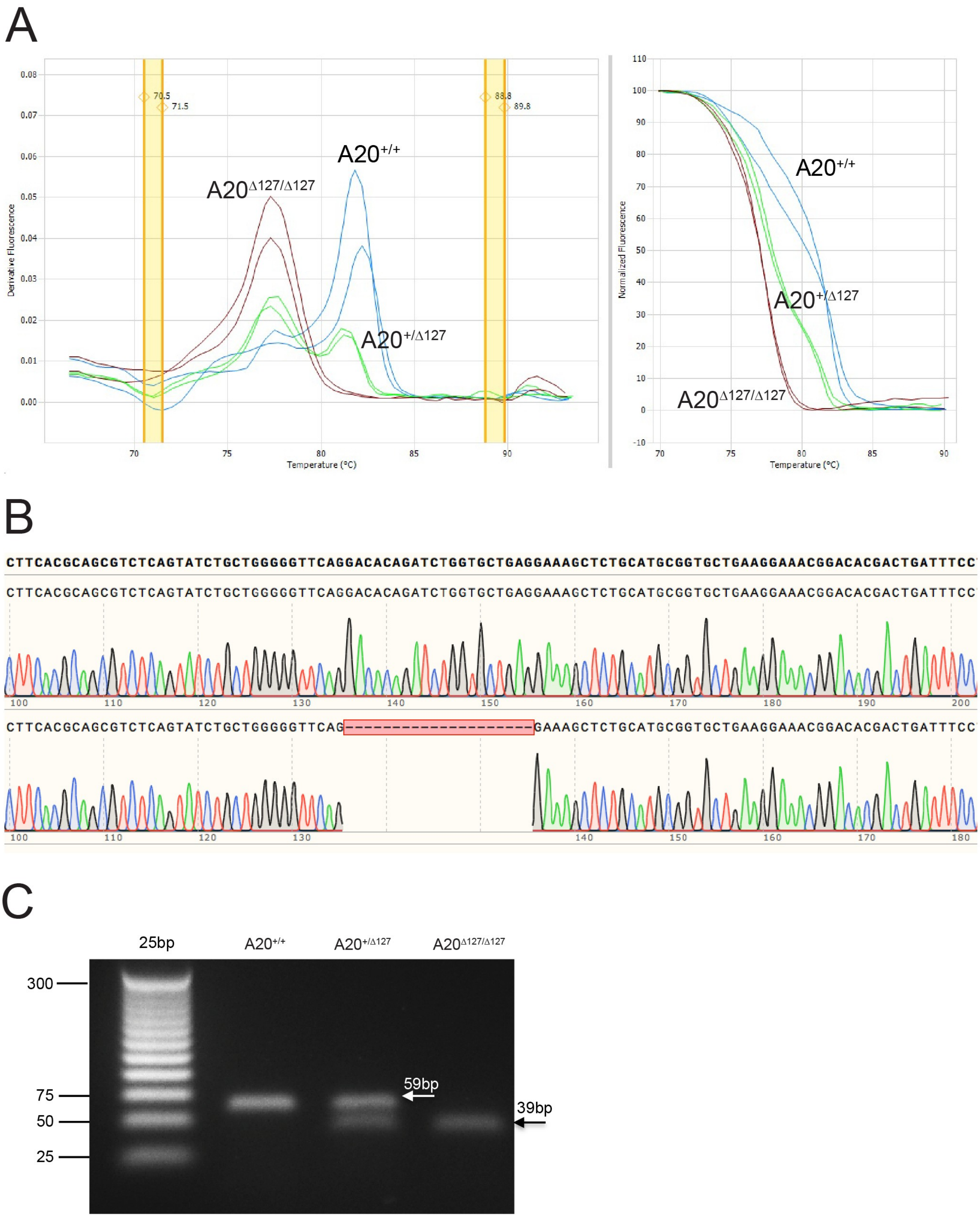

A
Derivative Fluorescence
Temperature (°C)
70.5
71.5
88.8
89.8
A20^{+/+}
A20^{Δ127/Δ127}
A20^{+/Δ127}
Normalized Fluorescence
B
CTTCACGCAGCGTCTCAGTATCTGCTGGGGGTTCAGGACACAGATCTGGTGCTGAGGAAAGCTCTGCATGCGGTGCTGAAGGAAACGGACACGACTGATTTCC
CTTCACGCAGCGTCTCAGTATCTGCTGGGGGTTCAGGACACAGATCTGGTGCTGAGGAAAGCTCTGCATGCGGTGCTGAAGGAAACGGACACGACTGATTTCC
CTTCACGCAGCGTCTCAGTATCTGCTGGGGGTTCAG------------------GAAAGCTCTGCATGCGGTGCTGAAGGAAACGGACACGACTGATTTCC
C
25bp
A20^{+/+}
A20^{+/Δ127}
A20^{Δ127/Δ127}
300
75
50
25
59bp
39bp

Figure S3

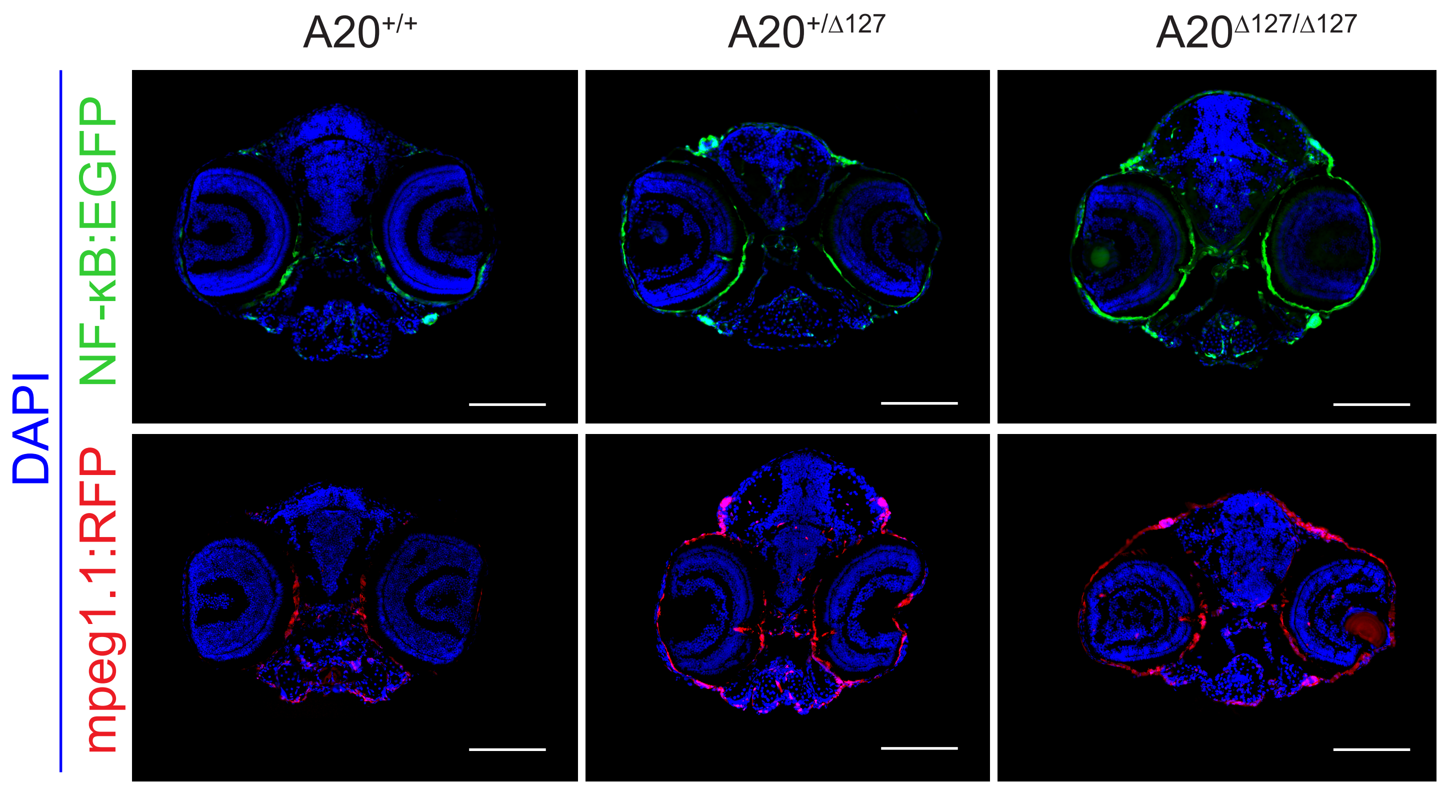

Figure S4

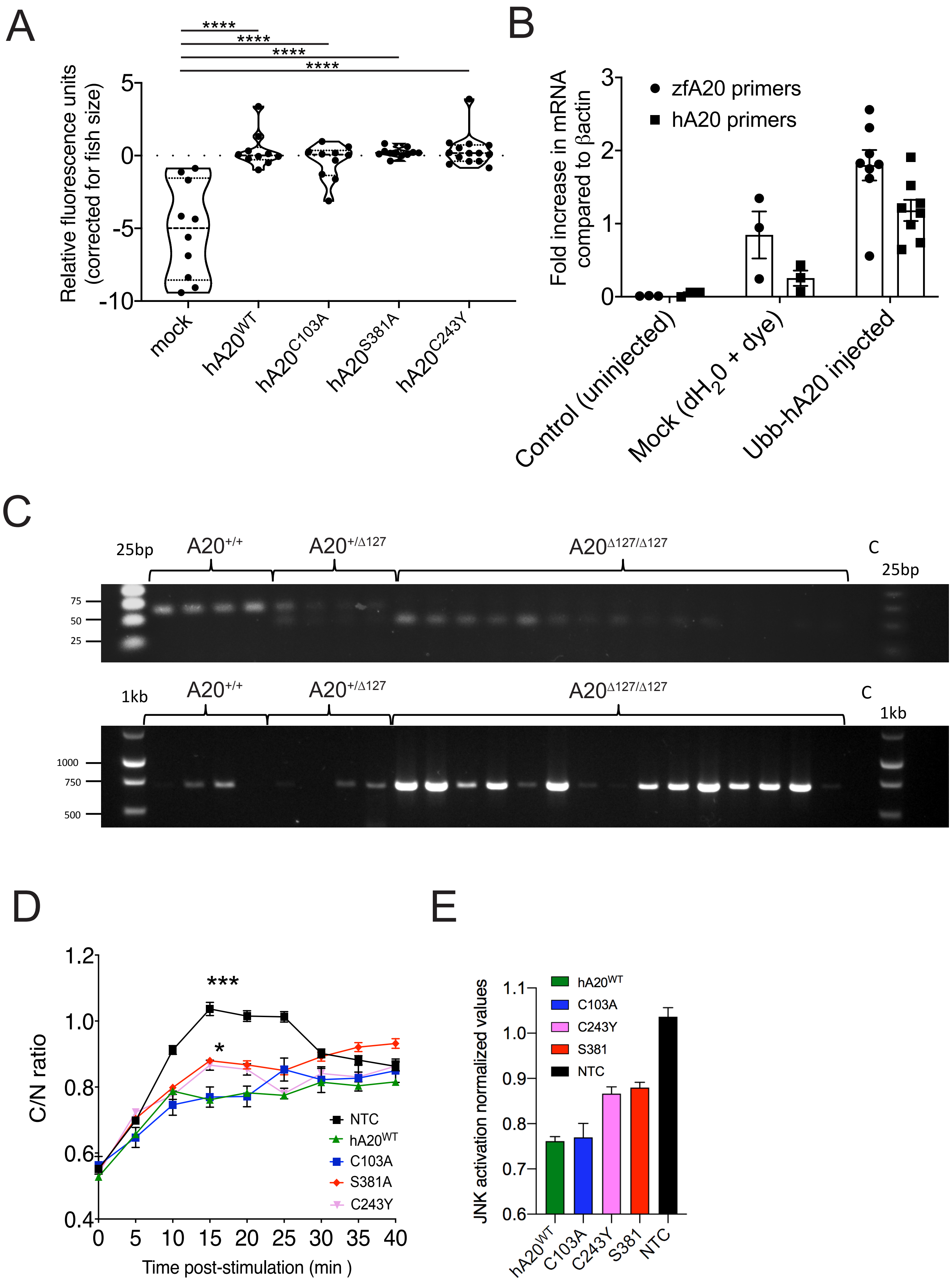
A
B
C
D
E
Relative fluorescence units (corrected for fish size)
****
mock
hA20WT
hA20C103A
hA20S381A
hA20C243Y
Fold increase in mRNA compared to βactin
zfA20 primers
hA20 primers
Control (uninjected)
Mock (dH20 + dye)
Ubb-hA20 injected
25bp
A20+/+
A20+/Δ127
A20Δ127/Δ127
C
1kb
C/N ratio
Time post-stimulation (min)
NTC
hA20WT
C103A
S381A
C243Y
JNK activation normalized values
S381

Figure S5
