# Supplementary material for: A zebrafish functional genomics model to investigate the role of human A20 variants in vivo
Source: Sci Rep. 2020 Nov 5;10:19085. doi: 10.1038/s41598-020-75917-6 (PMC7644770; doi:10.1038/s41598-020-75917-6)
Supplement: Supplementary file 6 — Supplementary Legends. [file 41598_2020_75917_MOESM6_ESM.docx]

**ONLINE SUPPLEMENTAL MATERIAL**

**Figure S1. Genomic structure of the TNFAIP3 (A20) locus.**

**A** HiC data (H1 cells) visualized over the A20 locus, overlapping A20 SNPs and H3K27ac ChIP-seq signal. Insets correspond to three evolutionarily-conserved (PhyloP, vertebrate conservation) A20 SNPs, which coincide with the regulatory H3K27ac mark. The alternating yellow and pink shaded areas are predicted Topologically Associated Domains (TADs), whereas the heatmap (white-red) represents normalized interaction frequency, as described in (Wang, Song et al. 2018). The SNP IDs for SNPs with insets in the figure are (a) chr6:138085249-138085249 (rs7753394), (b), chr6:138085366-138085366 (rs7773904), and (c), chr6:138132517-138132517 (rs10499197). **B** H3K27ac ChIP-seq (24 hpf embryos) visualized over the A20 locus. Boxes represent human-fish DNA sequence conservation. Software used for this figure: 3D genome browser (<http://promoter.bx.psu.edu/hi-c/>), University of California Santa Cruz Genome Browser ([genome.ucsc.edu](http://genome.ucsc.edu)), bowtie (<http://bowtie-bio.sourceforge.net/index.shtml>).

**Figure S2. Protein homology of TNFAIP3 across multiple vertebrate lineages**

The consensus sequence (cons) below each amino acid reports an “*” (asterisk) when all the amino acids are the same, a “:” (colon) indicates conservation between groups of strongly similar properties and a “.” (period) indicates conservation between groups of weakly similar properties. The amino acids comprising the OTU domain catalytic triad (Asp70, Cys103, His256) and position Ser381 are also shown. Note these two protein regions are explicitly conserved across species. Data alignment generated using ClustalOmega and edited to highlight regions of interest. Data alignment generated using ClustalOmega and edited to highlight regions of interest using Adobe Illustrator CC 2018 (<https://www.adobe.com/au/products/illustrator.html>).

**Figure S3. Validation of A20 deletion in TALEN targeted zebrafish**

**A** HRMA curves (derivative on the left and raw-melt on the right) showing the three possible genotypes arising from a cross between two A20^+/∆127^. The trace for a zebrafish carrying either A20 wild type alleles (Blue), or heterozygous (Green) or homozygous (Purple) for the mutant A20 allele are shown. **B** Sequence trace and alignment for a zebrafish carrying either the A20 wild type A20 allele or the deletion. **C** PCR amplification of the TALEN targeted region of the A20 gene for A20^+/+^, A20^+/∆127^ and A20^∆127/∆127^ zebrafish. The displayed gel has been cropped to improve clarity and conciseness of the figure. Software used for this Figure: Adobe Illustrator CC 2018 (<https://www.adobe.com/au/products/illustrator.html>), Illumina Eco Software v4.0.7.0 (<https://sapac.illumina.com/content/dam/illumina-marketing/documents/products/datasheets/datasheet_eco_system.pdf>), SnapGene v4.0.7 (<https://www.snapgene.com/>), Bio-rad ChemiDoc v6 (<https://www.bio-rad.com/en-au/product/chemidoc-imaging-system?ID=OI91XQ15>).

**Figure S4. Co-localisation of NF-κB and MPEG1 reporter signals in zebrafish**

Representative transversal zebrafish head cryosections for a NF-κB:EGFP and mpeg1.1:RFP zebrafish and counter stained with DAPI for each A20 genotype at 1 wpf. The scale bars represent 75 μm.

**Figure S5. Control Data for A20-dependent JNK inhibition and *in vivo* gene expression**

**A** Relative fluorescence units, corrected for fish size. No statistical significance was observed between groups of fish injected with different human A20-EGFP constructs. P-values were determined using ANOVA. All of the latter groups were statistically different (****P < 0.0001) when compared to the mock injected fish. **B** qPCR data showing human hA20 expression in injected zebrafish embryos but not control or mock injected zebrafish. **C** PCR data showing transgene expression (GFP) in surviving zebrafish 3wpf injected with the Ubb-hA20WT-GFP expression construct. Fish were from het x het crosses blindly injected with Ubb-hA20WT-GFP and genotyped for *tnfaip3* and GFP expression. Top panel shows zebrafish *tnfaip3* genotype and bottom panel shows GFP expression. All surviving A20 null zebrafish express GFP. **D** Impact of A20 variants in mammalian cells. JNK activation kinetics for HEK293 cells transiently non-transfected (NTC), or co-transfected with WT human-A20 or the C103A, S381A or C243Y mutant and treated with or without hTNFα for 1 hour and quantified at 5 minutes intervals. Data shows the ratio of Cytoplasm over Nuclear (C/N) JNK activation, represented as mean ± standard error. **E** Data from the 15 min time point for all A20 variants is represented in histogram format. Statistical analysis carried as Area Under the Curve (AUC) compared to WT hA20. WT and NTC = ***P < 0.001, WT and S381A or C243Y = *P < 0.05. The data on this graph have been generated by three independent experiments. Software used for this Figure: GraphPad Prism v7.0 (<https://www.graphpad.com/scientific-software/prism/>), Adobe Illustrator CC 2018 (<https://www.adobe.com/au/products/illustrator.html>), Bio-rad ChemiDoc v6 (<https://www.bio-rad.com/en-au/product/chemidoc-imaging-system?ID=OI91XQ15>).

**Video S1 and S2. 3-D projections of livers from wild type and A20**^∆127/∆127^ **zebrafish.**

Video files represent 3-D projections of confocal stacks showing liver images from (A) wild type (WT LIVER 3D) and (B) homozygous A20^∆12^ (HOM LIVER 3D) zebrafish respectively. Images were acquired with a Leica Confocal Microscope M205 FA and reconstructed from z-stacks using IMARIS Image Analysis Software v8 (BITPLANE, Switzerland) (<https://imaris.oxinst.com/>).

**Video S3. 3-D rendering of an A20 Two-Photon microscope tilemap of a double-reporter (NF-κB:EGFP;mpeg1.1:RFP) A20^+/+^.**

The video shows three channels: Collagen (in magenta) generated through Second Harmonic, NF-κB (in green) generated though EGFP fluorescence and mpeg1.1 (in red) generated through RFP fluorescence. The video navigates through the 3D rendering of a double-positive NF-κB:EGFP;mpeg1.1:RFP) A20^+/+^ zebrafish. It switches between the overlapping of the green and red signals and the individual mpeg1.1 red channel to better show the colocalization of NF-κB in macrophages in a more cell-sparse area of this representative zebrafish. Software used for this Figure: IMARIS Image Analysis Software v8 (BITPLANE, Switzerland) (<https://imaris.oxinst.com/>).

**Video S4. Two-Photon z-stack 15 minutes timelapse of a double reporter NF-κB:EGFP;mpeg1.1:RFP A20;A20^+/+^ zebrafish.**

The red track shows the path taken by a macrophage (in yellow) expressing both RFP and GFP. The video is reproduced in loop and changed perspective, as well as overlaying/single channels, to offer a better sense of the motility of these cells within the immobilized live zebrafish. Software used for this Figure: IMARIS Image Analysis Software v8 (BITPLANE, Switzerland) (<https://imaris.oxinst.com/>).
